# Supplementary material for: USP26 functions as a negative regulator of cellular reprogramming by stabilising PRC1 complex components
Source: Nat Commun. 2017 Aug 24;8:349. doi: 10.1038/s41467-017-00301-4 (PMC5571198; doi:10.1038/s41467-017-00301-4)
Supplement: Supplementary file 1 — Supplementary Information [file 41467_2017_301_MOESM1_ESM.pdf]

Title of file for HTML: Supplementary Information
Description: Supplementary Figures, Supplementary Table

Title of file for HTML: Supplementary Data
Description: Relative expression of the 90 marker genes during reprogramming of Usp26 shRNA and control shRNA lentivirus-transduced OSKM-MEFs, Table related to main Figure 3.

Title of file for HTML: Peer Review File
Description:

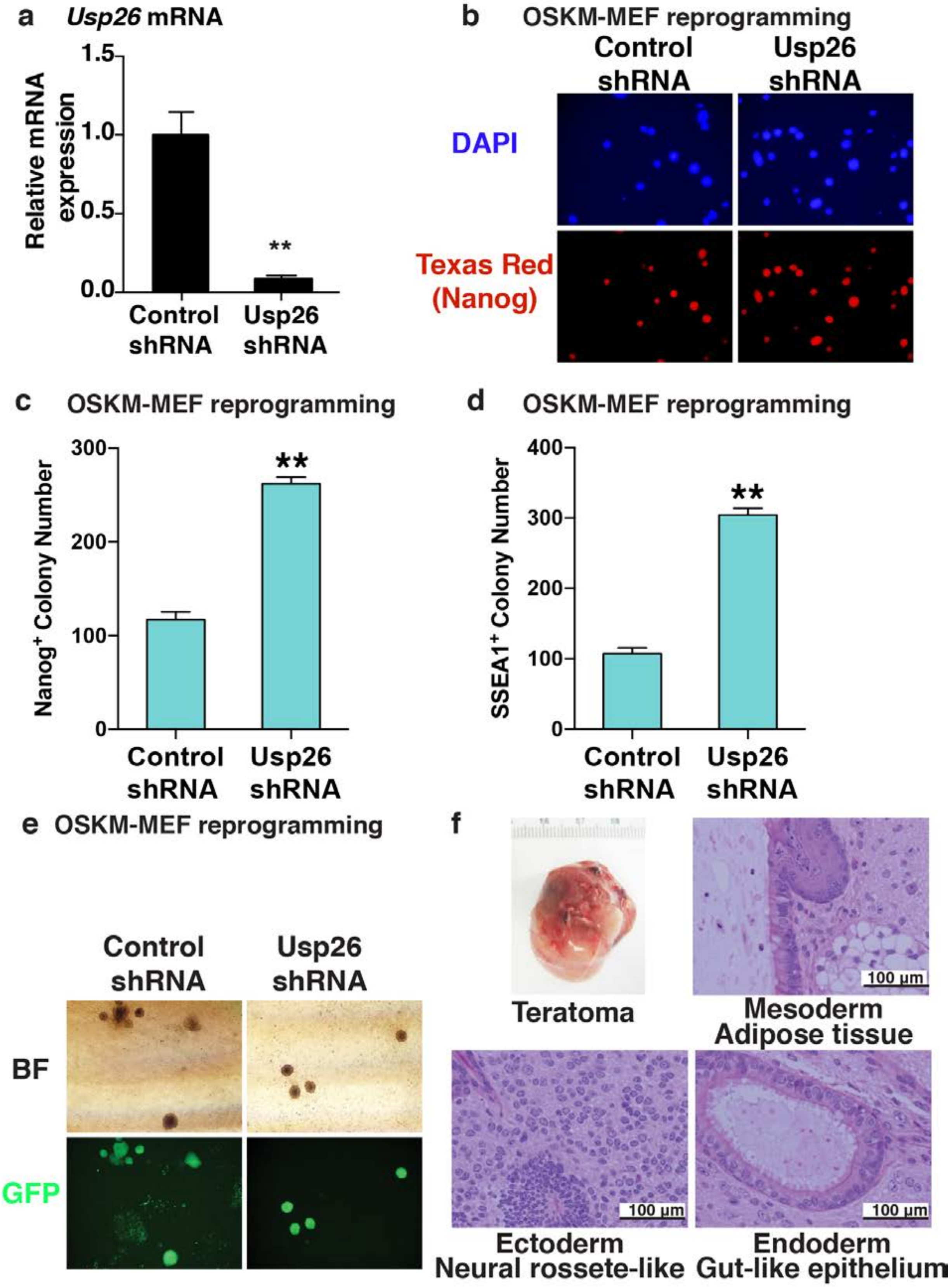
a
Usp26 mRNA
Relative mRNA expression
1.5
1.0
0.5
0.0
**
Control shRNA
Usp26 shRNA
b
OSKM-MEF reprogramming
Control shRNA
Usp26 shRNA
DAPI
Texas Red (Nanog)
c
OSKM-MEF reprogramming
Nanog+ Colony Number
300
200
100
0
**
Control shRNA
Usp26 shRNA
d
OSKM-MEF reprogramming
SSEA1+ Colony Number
400
300
200
100
0
**
Control shRNA
Usp26 shRNA
e
OSKM-MEF reprogramming
Control shRNA
Usp26 shRNA
BF
GFP
f
Teratoma
100 µm
Mesoderm
Adipose tissue
100 µm
Ectoderm
Neural rossete-like
100 µm
Endoderm
Gut-like epithelium

Supplementary Figure 1. Characterization of *Usp26* shRNA knockdown in iPSCs, related to Figure 1 in the main text

(a) qPCR analysis of *Usp26* shRNA knockdown efficiency, ** $p<0.01$ compared to control shRNA.

(b) Immunofluorescence microscopic images of Nanog in *Usp26* knockdown iPSCs.

(c)& (d) Quantification of $Nanog^+$ (c) and SSEA1 (d) colonies after 12 days of OSKM induction in MEFs transduced with control or *Usp26* shRNA, ** $p<0.01$ compared to control shRNA, n=4.

(e) Microscopic images of iPSC morphology and GFP immunofluorescence after *Usp26* knockdown.

(f) Gross morphology and histology of *Usp26* knockdown iPSC teratoma assay.

The data are presented as means ± SD from three independent experiments; (a, c, d) Unpaired two-tailed Student's t-test.

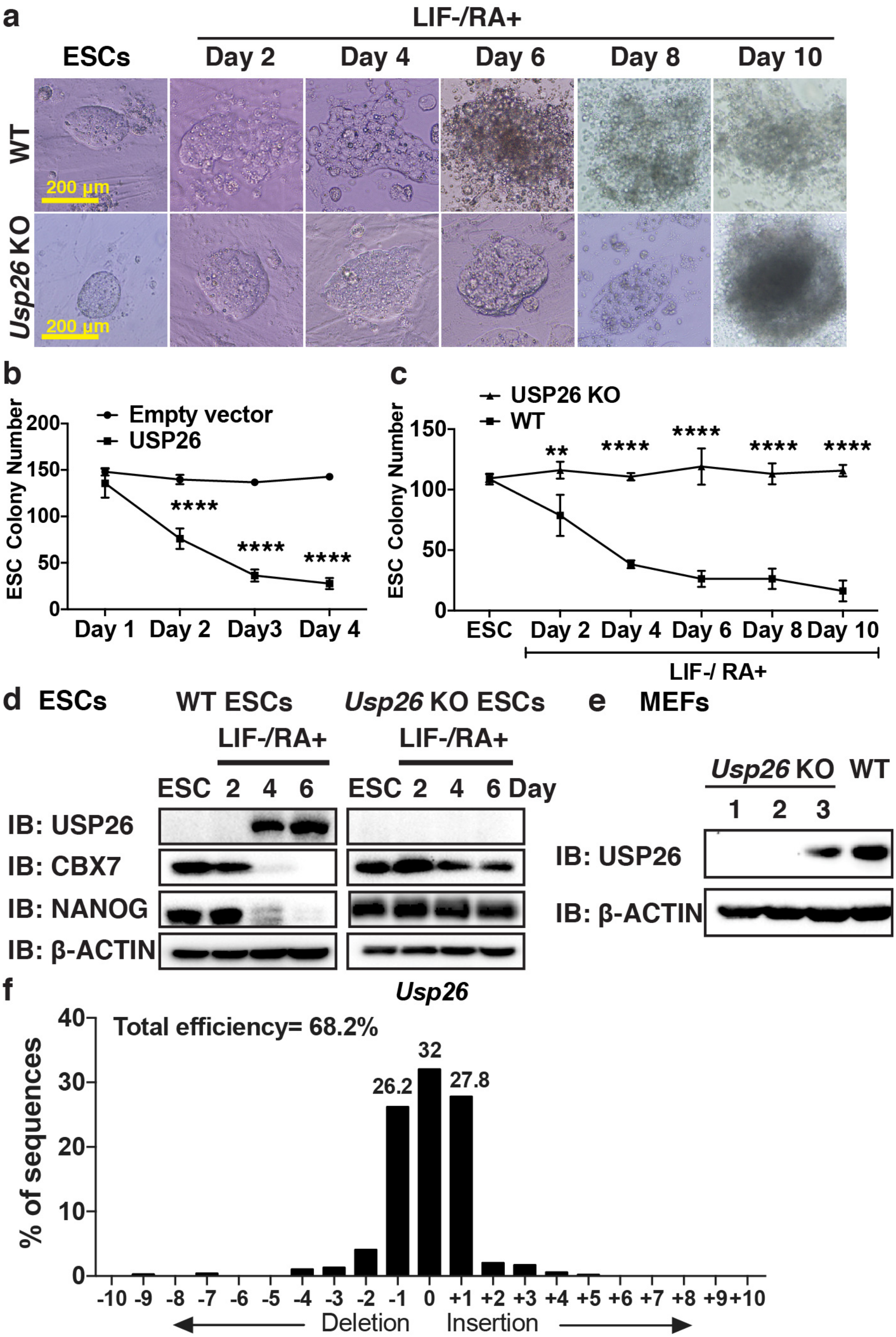

a
LIF-/RA+
ESCs
Day 2
Day 4
Day 6
Day 8
Day 10
WT
Usp26 KO
200 μm
200 μm
b
Empty vector
USP26
ESC Colony Number
Day 1
Day 2
Day3
Day 4
****
c
USP26 KO
WT
ESC Colony Number
ESC
LIF-/ RA+
**
****
d ESCs
WT ESCs
Usp26 KO ESCs
ESC 2 4 6 Day
IB: USP26
IB: CBX7
IB: NANOG
IB: β-ACTIN
e MEFs
Usp26 KO
WT
1 2 3
f
Usp26
Total efficiency= 68.2%
% of sequences
26.2
32
27.8
Deletion
Insertion

Supplementary Figure 2. *Usp26* knockout delays RA-induced ESC differentiation and maintains ESC morphology, related to Figure 2 in the main text

(a) Brightfield microscopic images of ESCs during LIF withdrawal (LIF-) and RA-induced (RA+) differentiation in wild-type (WT) and Usp26 knockout (KO) ES cells. WT or Usp26 KO ESCs were cultured in ES differentiation medium (LIF withdrawal with 1 μM RA). Individual colonies were tracked and taken pictures at days 0, 2, 4, 6, 8 and 10 under microscope.

(b) Quantification of ESC colonies transduced with Dox inducible GFP-tagged m*Usp26* overexpression or with GFP-tagged empty vector, **** $p<0.0001$ compared to empty vector.

(c) Quantification of ESC colonies during LIF withdrawal (LIF-) and RA-induced (RA+) differentiation in wild-type (WT) and Usp26 knockout (KO) ES cells, ** $p<0.01$, **** $p<0.0001$ compared to WT.

(d) Western blot analysis of protein levels of pluripotency markers in wild-type (WT) or Usp26 knockout (KO) ES cells differentiated by LIF withdrawal (LIF-) and RA-induction (RA+).

(e) Western blot analysis of CRISPR/Cas9 knockout for USP26 in MEF cells.

(f) TIDE assay of CRISPR/Cas9 knockout in *Usp26* in MEF cells.

The data are presented as means ± SD from three independent experiments, (b, c) Two-way ANOVA for multiple comparisons.

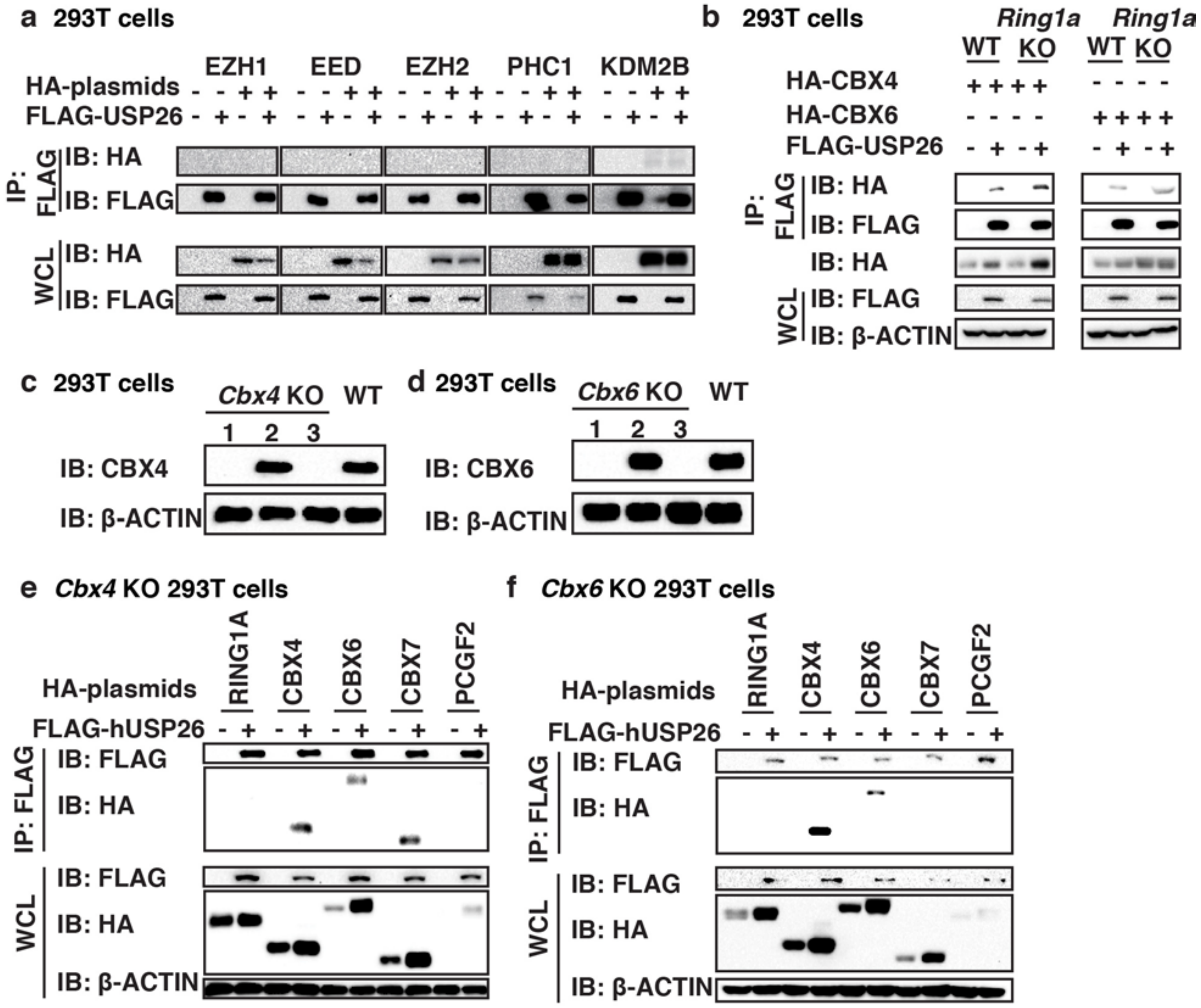

Supplementary Figure 3. USP26 interacts with CBX4 and CBX6, related to Figure 4 in the main text

(a) 293T cells were transfected with HA-tagged EZH1, EED, EZH2, PHC1, and KDM2B plus FLAG-hUSP26 (+) or empty vector (-). Following IP with anti-FLAG beads, specific proteins were analyzed by western blotting using anti-HA antibody.

(b) Wild-type (WT) or RING1A knockout (KO) 293T cells were transfected with HA-tagged CBX4 or CBX6 plus FLAG-hUSP26. Following IP with anti-FLAG beads, specific proteins were analyzed by western blotting using anti-HA antibody.

(c) Western blot analysis of CRISPR/Cas9 knockout of CBX4 in 293T cells.

(d) Western blot analysis of CRISPR/Cas9 knockout of CBX6 in 293T cells.

(e) CBX4 knockout 293T cells were transfected with HA-tagged RING1A, CBX4, CBX6, CBX7, or PCGF2 plus FLAG-hUSP26. Following IP with anti-FLAG beads, specific proteins were analyzed by western blotting using anti-HA antibody.

(f) CBX6 knockout 293T cells were transfected with HA-tagged RING1A, CBX4, CBX6, CBX7, or PCGF2 plus FLAG-hUSP26. Following IP with anti-FLAG beads, specific proteins were analyzed by western blotting using anti-HA antibody.

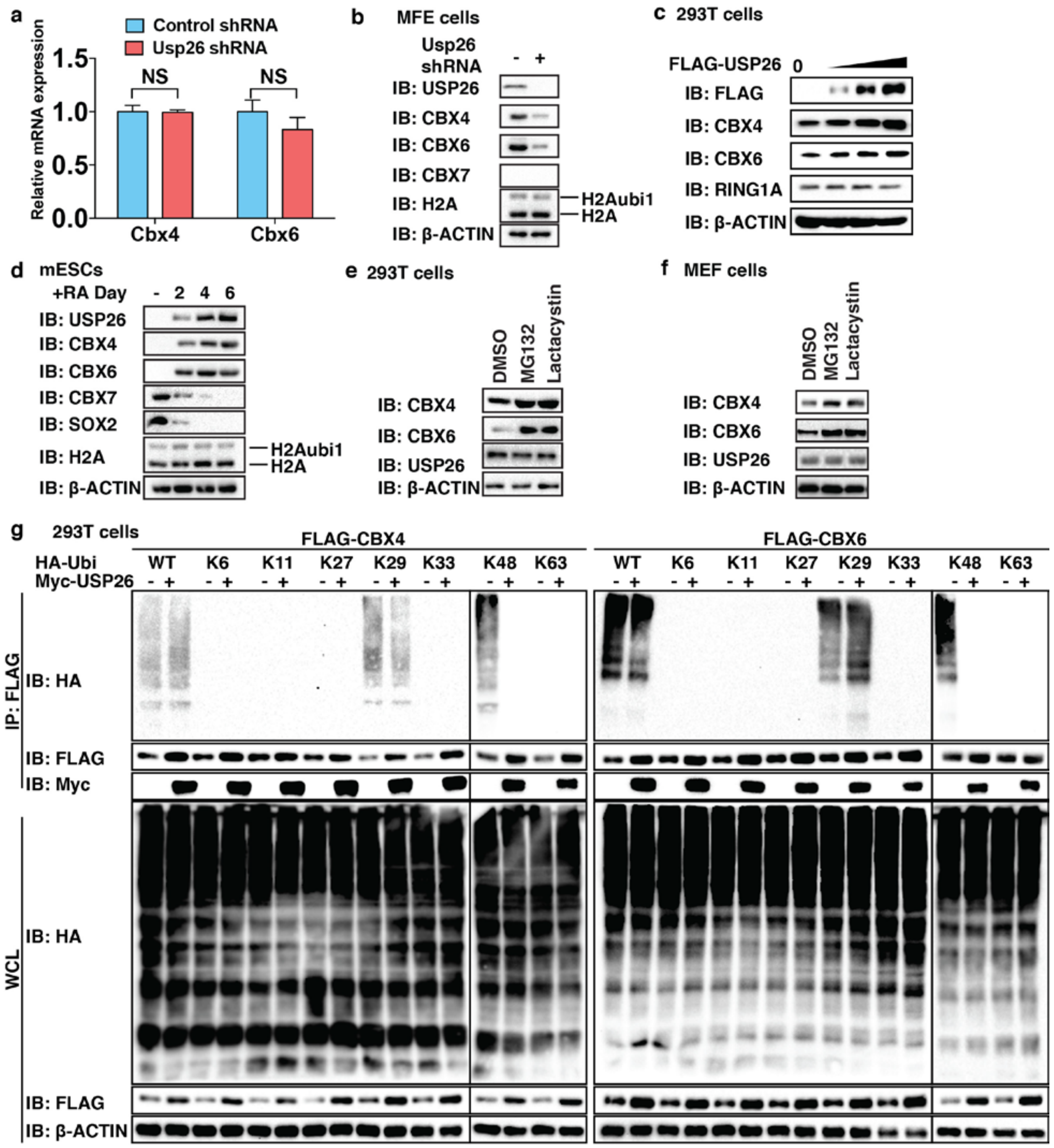

Supplementary Figure 4. USP26 specifically removes K48-linked polyubiquitin chain and blocks degradation of CBX4 and CBX6, related to Figure 5 in the main text

(a) MEFs were transduced with mouse *Usp26* shRNA or control shRNA lentivirus, cell extracts were harvested, and mRNA levels were analyzed by qPCR. The data are presented as means ± SD from three independent experiments.

(b) MEFs were transduced with mouse *Usp26* shRNA or control shRNA lentivirus, cell extracts were harvested, protein levels were analyzed by western blotting with specific antibodies as indicated

(c) 293T cells were transfected with different amounts of FLAG-USP26 (0, 0.2 μg, 0.5 μg, and 1 μg). Cell extracts were harvested and analyzed by western blotting with specific antibodies, as indicated.
(d) Mouse ES cells were infected with or without RA treatment, and cell extracts were harvested at different time points, protein levels were analyzed by western blotting using specific antibodies, as indicated.
(e) 293T cells were treated with DMSO, MG132 or Lactacystin. Specific proteins were analyzed by western blotting using anti-CBX4 and anti-CBX6 antibodies. β-actin was used as a loading control.
(f) MEF cells with or without Usp26 lentivirus transduction were treated with DMSO, MG132 or Lactacystin. Specific proteins were analyzed by western blotting using anti-CBX4, anti-CBX6 and anti-USP26 antibodies. β-ACTIN was used as a loading control.
(g) 293T cells were transfected with HA-tagged WT-, K6-, K11-, K27-, K29-, K33-, K48-, and K63-linked Ub, FLAG-CBX4 or FLAG-CBX6 with (+) or without (-) Myc-tagged USP26. Following IP with anti-FLAG beads, ubiquitination of CBX4 or CBX6 was analyzed by western blotting using anti-HA antibody.
(a) Unpaired two-tailed Student's t-test.

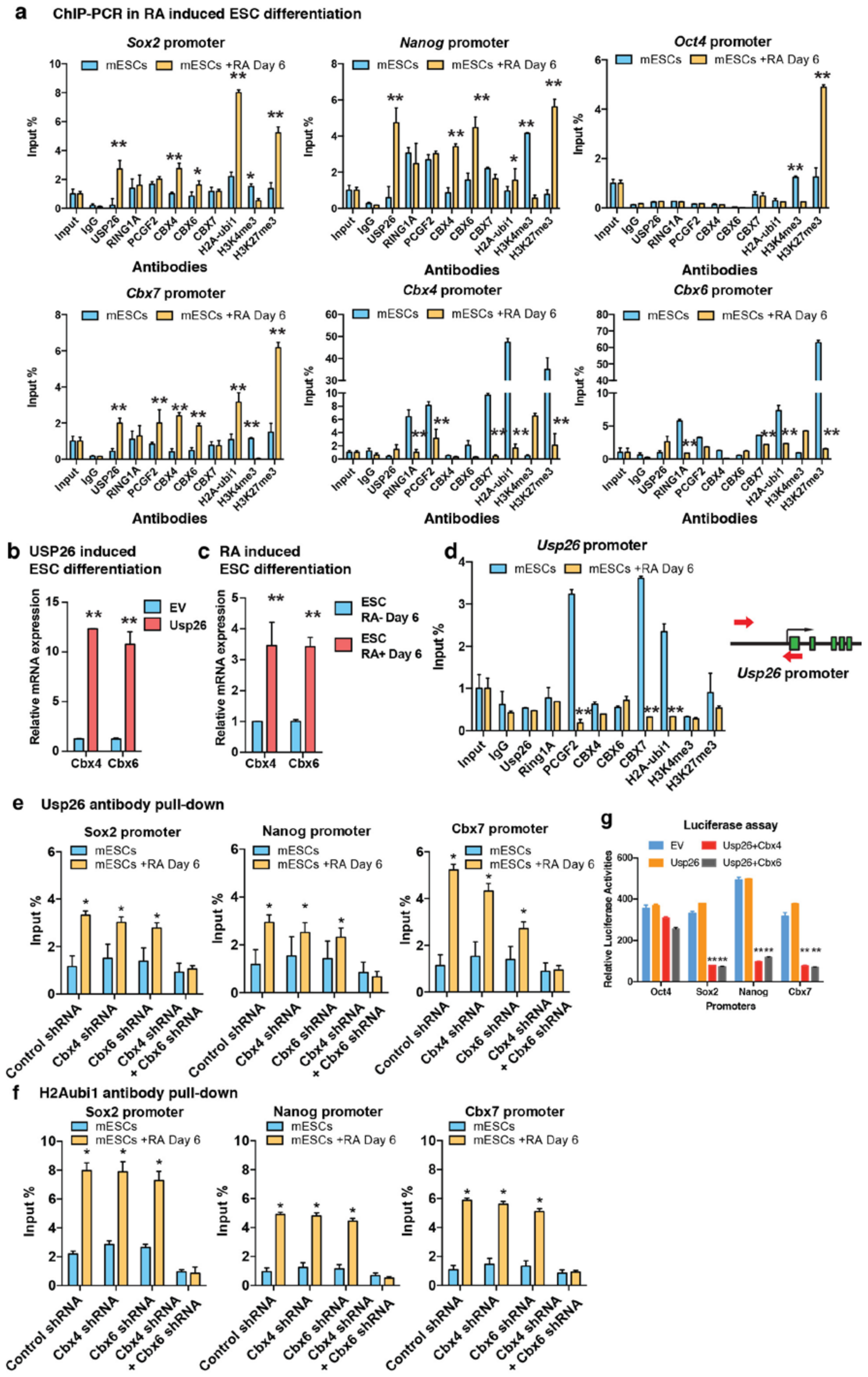

a ChIP-PCR in RA induced ESC differentiation
Sox2 promoter
Nanog promoter
Oct4 promoter
Cbx7 promoter
Cbx4 promoter
Cbx6 promoter
mESCs
mESCs +RA Day 6
Input %
Antibodies
Input
IgG
USP26
RING1A
PCGF2
CBX4
CBX6
CBX7
H2A-ubi1
H3K4me3
H3K27me3
b USP26 induced ESC differentiation
EV
Usp26
Relative mRNA expression
Cbx4
Cbx6
c RA induced ESC differentiation
ESC RA- Day 6
ESC RA+ Day 6
d Usp26 promoter
Usp26 promoter
e Usp26 antibody pull-down
Sox2 promoter
Nanog promoter
Cbx7 promoter
Control shRNA
Cbx4 shRNA
Cbx6 shRNA
Cbx4 shRNA + Cbx6 shRNA
f H2Aubi1 antibody pull-down
g Luciferase assay
EV
Usp26
Usp26+Cbx4
Usp26+Cbx6
Relative Luciferase Activities
Oct4
Sox2
Nanog
Cbx7
Promoters

Supplementary Figure 5. Accumulated CBX4 and CBX6 bind to pluripotent gene promoters during ESC differentiation, related to Figure 6 in the main text

(a) ChIP-PCR analysis of *Sox2*, *Nanog*, *Oct4*, *Cbx7*, *Cbx4*, and *Cbx6* promoters in ESC differentiation upon LIF withdrawal and treatment with RA, ** $p<0.01$ compared to day 0.

(b) qPCR analysis of mouse *Cbx4* and *Cbx6* mRNA expression in ESCs transduced with mouse *Usp26* or empty vector (EV), ** $p<0.01$ compared to EV at day 6.

(c) qPCR analysis of mouse *Cbx4* and *Cbx6* mRNA expression in ESCs induced with RA or without RA, ** $p<0.01$ compared to RA- treatment at day 6.

(d) ChIP-PCR analysis of *Usp26* promoters in ESC differentiation upon LIF withdrawal and treatment with RA, ** $p<0.01$ compared to day 0.

(e-f) ChIP-PCR analysis of *Sox2*, *Nanog and Cbx7* promoters in control shRNA, Cbx4 shRNA Cbx6 shRNA or Cbx4 and Cbx6 shRNA transduced ESCs, which were cultured upon LIF withdrawal and treatment with RA, * $p<0.5$, ** $p<0.01$ compared to day 0.

(g) Luciferase assay of *Oct4*, *Sox2*, *Nanog*, and *Cbx7* promoters with empty vector (EV), *Usp26*, or *Usp26* and *Cbx4 or Cbx6* co-transfection, ** $p<0.01$ compared to EV.

Red arrows indicate ChIP-PCR targets and black arrows indicate transcription start sites (TSSs) at the *Usp26* promoters. The data are presented as means ± SD from three independent experiments (a-f) Unpaired two-tailed Student's t-test; (g) Two-way ANOVA for multiple comparisons.

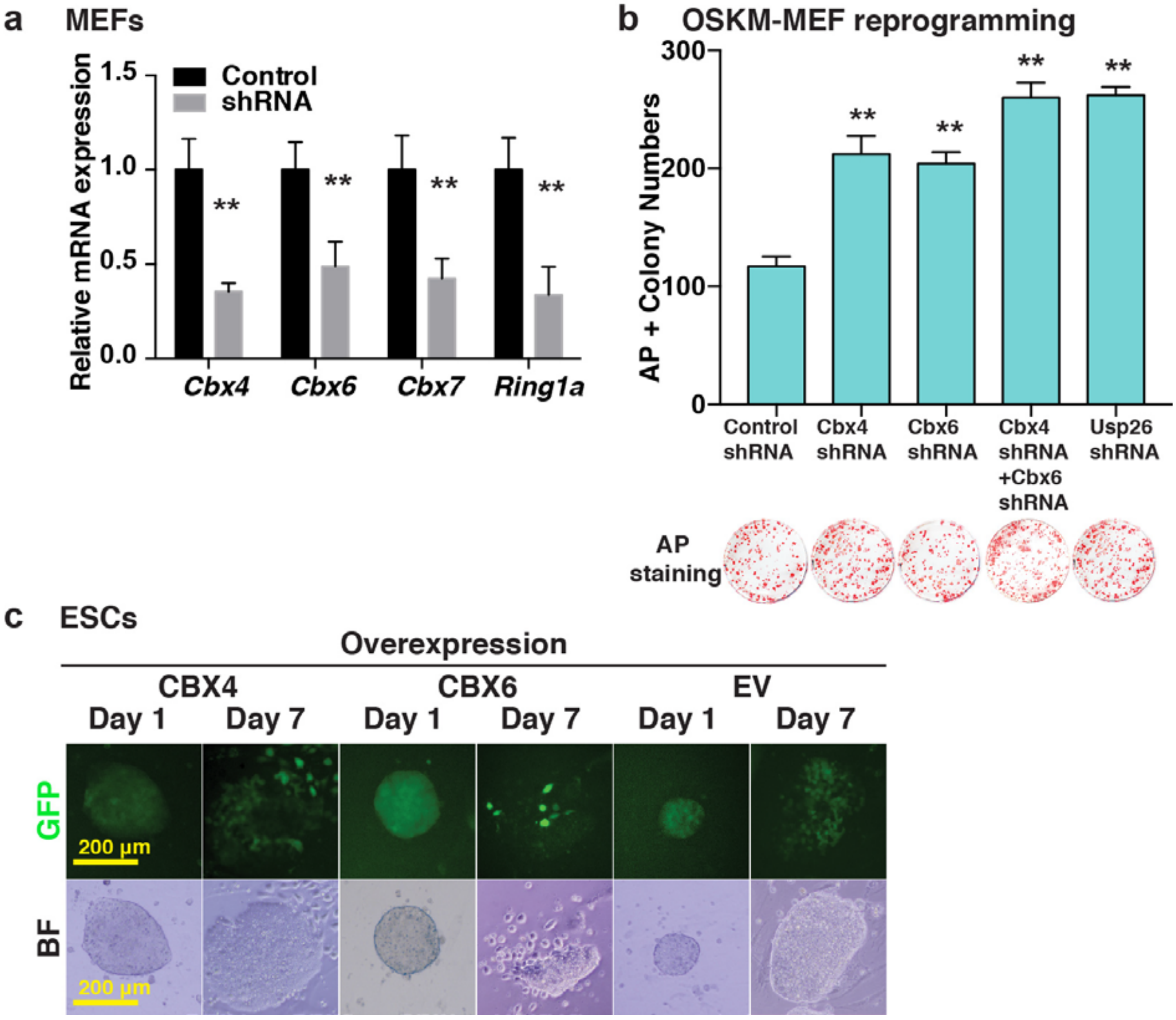

Supplementary Figure 6. CBX4 and CBX6 promote ESC differentiation, related to Figure 7 in the main text

(a) qPCR analysis of *Cbx4*, *Cbx6*, *Cbx7*, and *Ring1a* shRNA knockdown efficiency. (** $p<0.01$ compared to control shRNA).

(b) AP staining of iPSC colonies after OSKM induction in MEF cells infected with *Usp26*, *Cbx4*, *Cbx6*, or control shRNA lentivirus. OSKM transgenic MEF cells were transduced with GIPZ lentivirus-based shRNAs targeting Cbx4, Cbx6, or Cbx4 and Cbx6 and selected with puromycin for 2 days. 1000 puromycin selected OSKM transgenic MEF cells were reseeded onto feeder cells in 6-well plates. The next day, modified iSF1 medium containing 2 μg/ml Dox was added and replenished every day. The efficiency of iPSC formation was calculated based on the number of AP^+ iPSC colonies. The data are presented as means ± SD from four independent experiments (** $p<0.01$ compared to control shRNA).

(c) Microscopic images of GFP and morphology, using brightfield (BF), of *Usp26* KO ESCs at day 1 and day 7 after transfection with Dox inducible GFP-m*Cbx4*, GFP-m*Cbx6*, or empty GFP vector (EV).

The data are presented as means ± SD from three independent experiments, (a) Unpaired two-tailed Student's t-test; (b) Two-way ANOVA for multiple comparisons.

Figure 2b

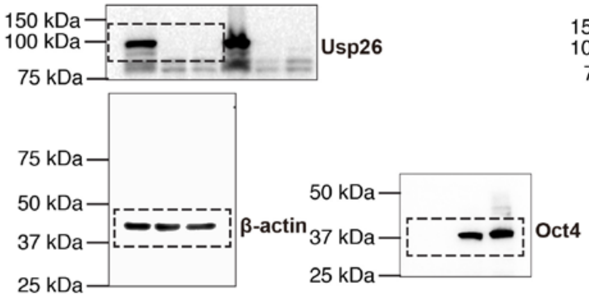

Figure 2e

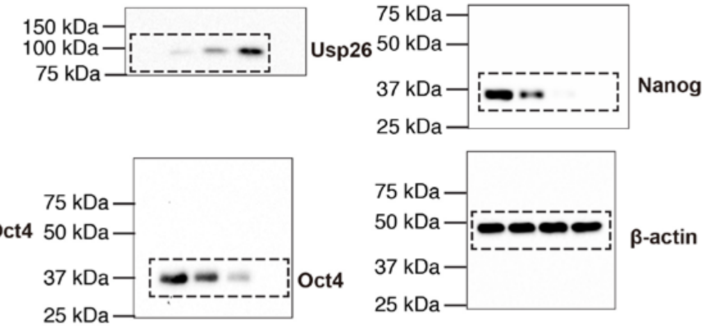

Figure 4a

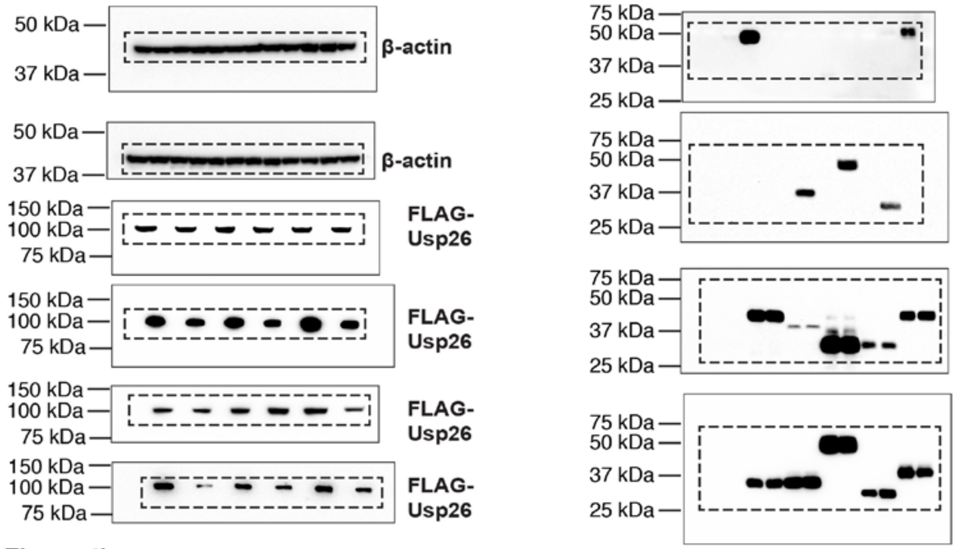

Figure 4b

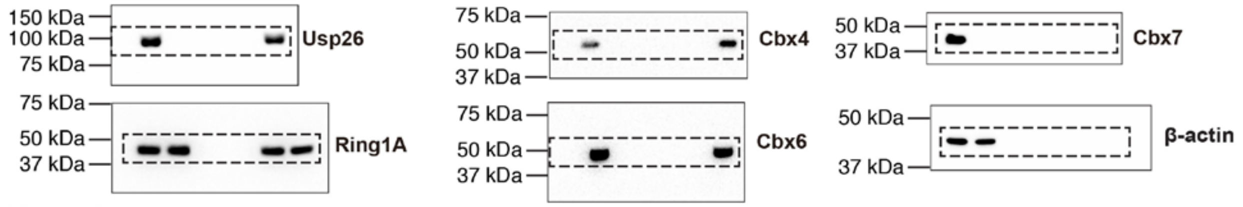

Figure 4c

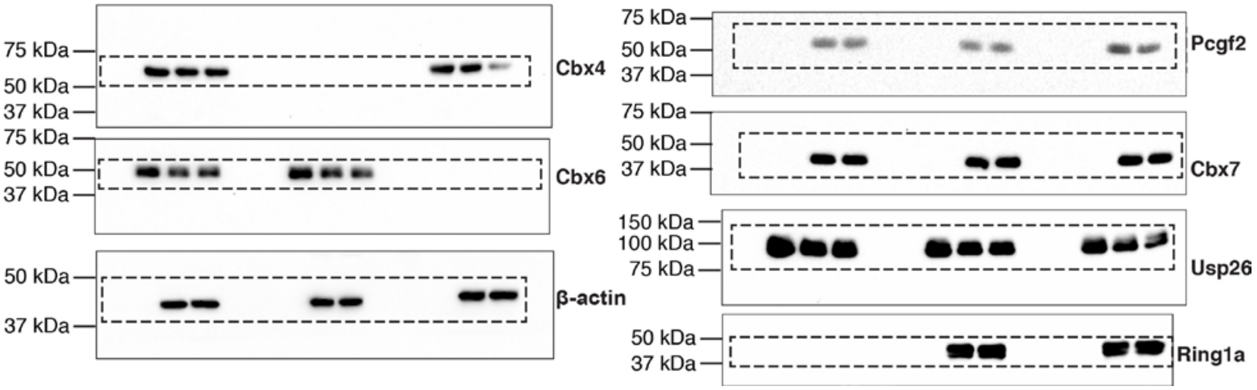

Figure 5b

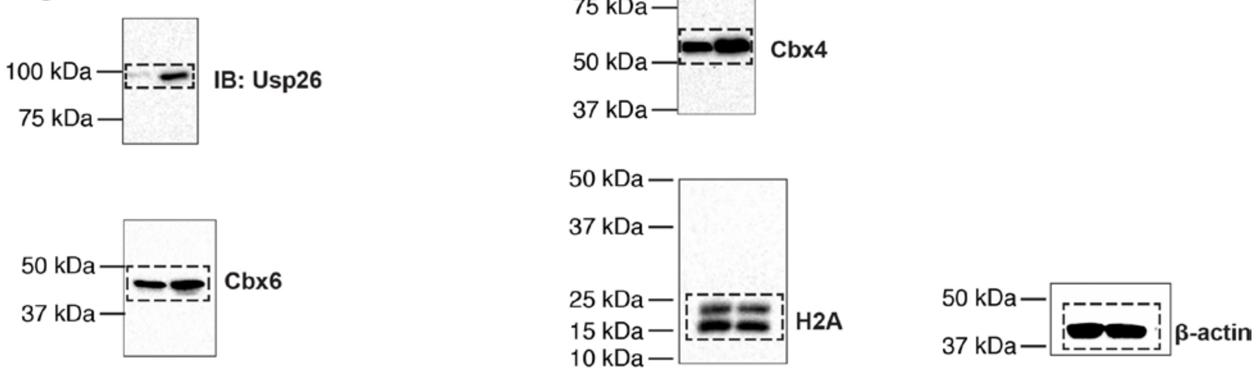

Figure 5d

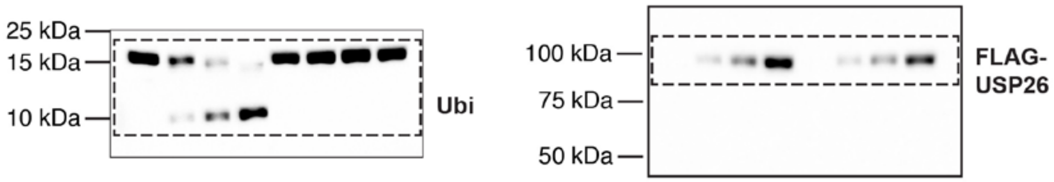

Figure 5c

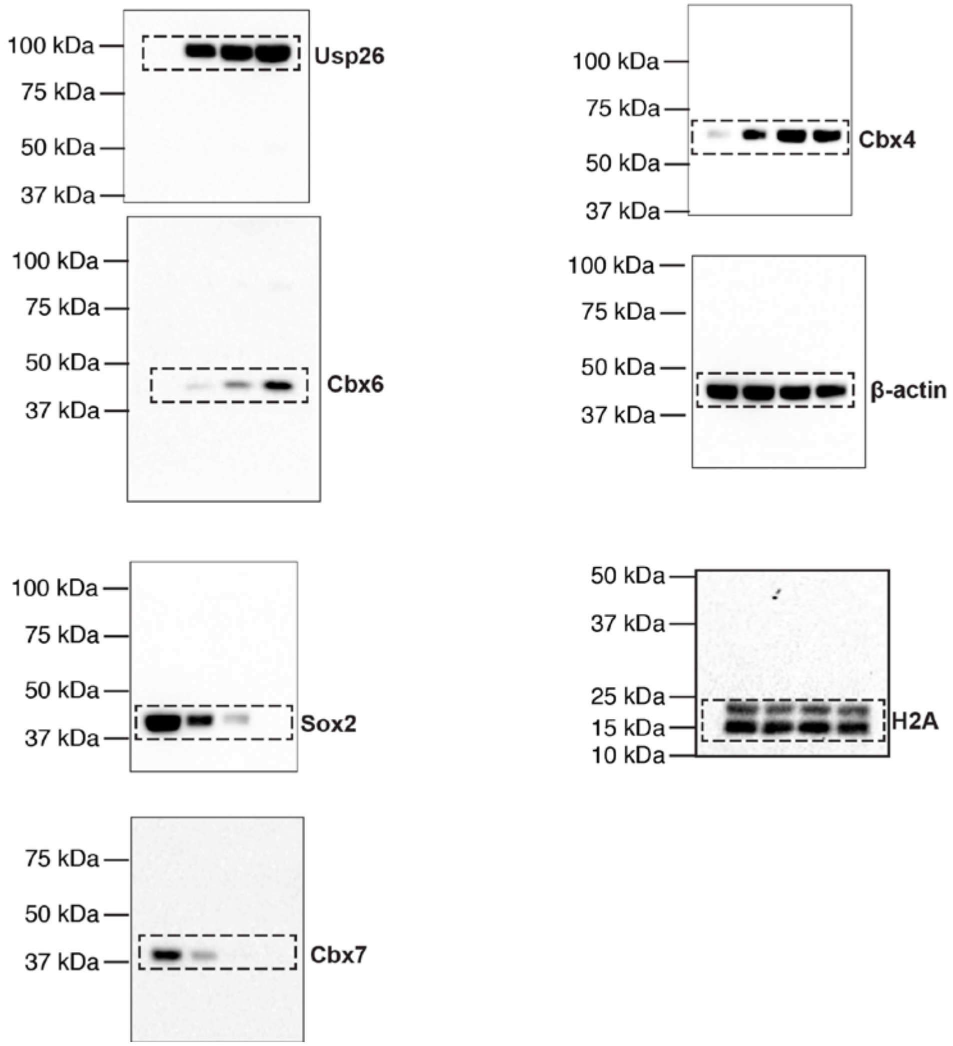

Figure 5e

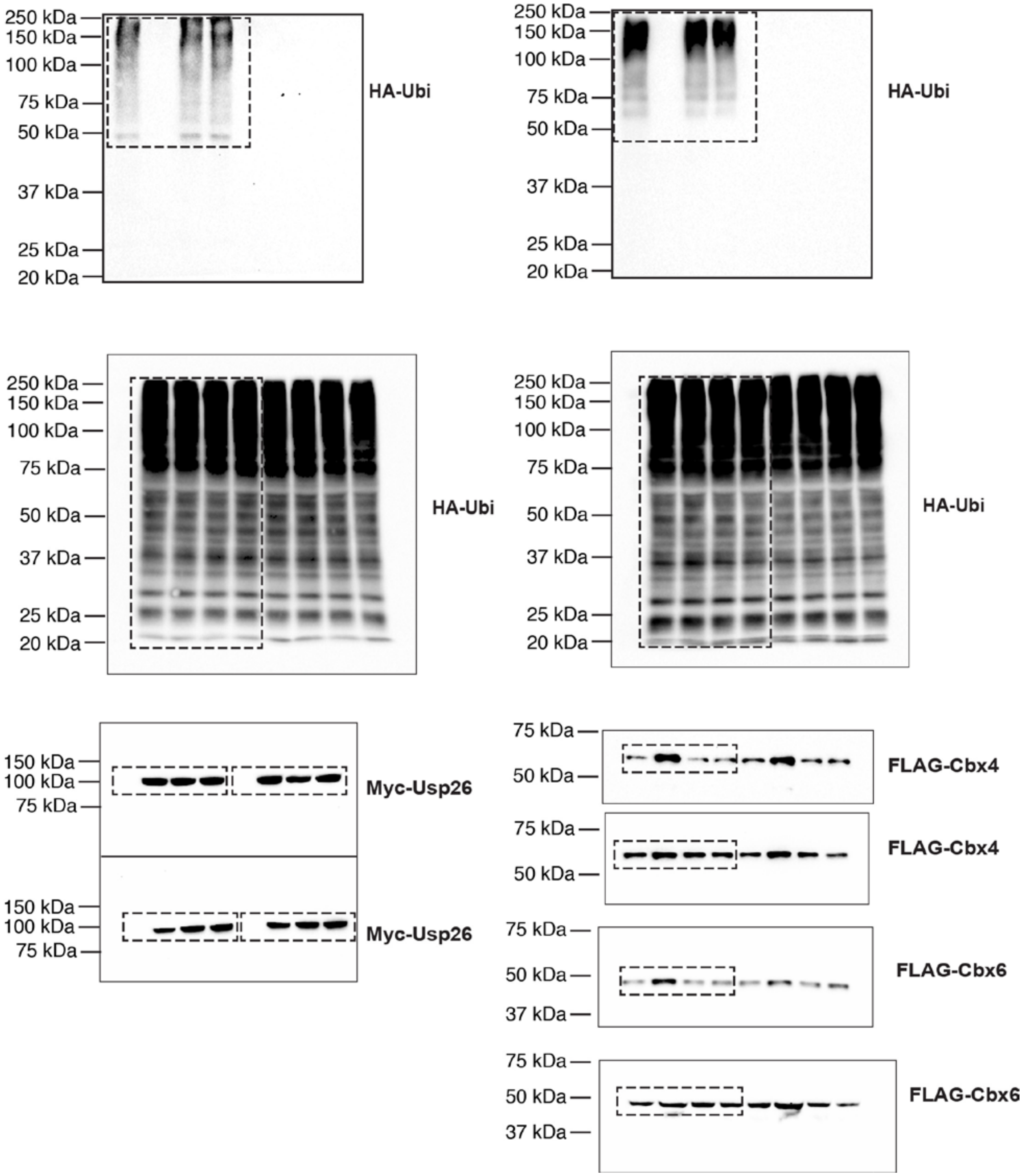

Supplementary Figure 7. Original western blots
The blots areas within boxes are shown in the figures, as indicated

Supplementary Table 1. Primers for RT-qPCR, ChIP-qPCR cloning and sequencing, and guide RNA used in this study. Table related to main Figure 1-7.

Gene	Forward	Reverse
**qPCR (Mouse)**		
Oct4	TGGGCTAGAGAAGGATGTGGT	GGAAAGGTGTCCCTGTAGCC
Sox2	GCCCTGCAGTACAACTCCAT	GACTTGACCACAGAGCCCAT
Klf4	TGTGACTATGCAGGCTGTGG	AGGTTTCTCGCCTGTGTGAG
Myc	ACGGAGTCGTAGTCGAGGTC	AGAGCTCCTCGAGCTGTTTG
Nanog	AAGCAGAAGATGCGGACTGT	ATCTGCTGGAGGCTGAGGTA
Esrrb	TGAAGGAGCCGCAACTAGAG	GCTGGAACACCTGAGGGTAA
Lin28	CTTCGGAACCCTTCCATGT	TCGGCTTCCTGTCTATGACC
Fbxl10	GAGGTGTGGATGGCAGTCTT	CCAACTGAGGTCAAGGGAGA
Tbx3	GAGGCCAAGGAACTTTGGGA	AGGGAACATTCGCCTTCCTG
Dppa2	CGGATGGCAAGAAAGTTGAGG	TTCTGGCCTCCCGAGATGTA
Sall4	CAGGGGAGTTCACTGGAGC	AGCACATCAACTGGGAGGAG
Utf1	GGATGTCCCGGTGACTACGTCTG	GGCGGATCTGGTTATCGAAGGGT
Gdf3	GTTCCAACCTGTGCCTCGCGTCTT	AGCGAGGCATGGAGAGAGCGGAGCA G
Grb2	TGCCACAGCAGCCAACCTACG	AACATGCCGGTCTGCCCGTG
Slc2a1	GGCCTGACTACTGGCTTTGT	TAAGCCAAACACCTGGGCAA
Nr6a1	CATCAGCCAAACCGCTTTCC	TTCACCATCTTGCCTGCGAT
Rex1	CCCTCGACAGACTGACCCTAA	TCGGGGCTAATCTCACTTTCAT
Eras	TGCCTACAAAGTCTAGCATCTTG	CTTTTACCAACACCACTTGCAC
Esg1	GAAGTCTGGTTCCTTGGCAGGATG	ACTCGATACACTGGCCTAGC
Gata4	CCATCTCGCCTCCAGAGT	CTGGAAGACACCCCAATCTC
Gata6	TGTAGAGGCCGTCTTGACCT	TACACAAGCGACCACCTCAG
Gata5	CAGTTGCCGCTGATTCCTCA	GGGTAGGTTGTGGGGTACGA
Foxp1	CCTCGCTCAAGGCATGATTC	GTGCTGGTCAGGTCTAGGC
Zfp532	TCAGGAAAGTTTACGCCTGCT	GGTCCTTGATCCCGTGCAT
Fut4	TAAGGCGCTGGTACTACGC	GGGACGAGAACCTACGGTC
Cripto	CAGTGCGTTTGAATTTGGACC	GCACGAACTGGAAAGACCGA
Tcl1	GTGTACTTGGATGAGTTTCGTCG	TTGCCACATTAAAGGCAGCTC
Nr5a2	TGAGGAACAACTCCGGGAAAA	CAGACACTTTATCGCCACACA
Klf2	CTCAGCGAGCCTATCTTGCC	CACGTTGTTTAGGTCCTCATCC
Tcf1	GACCTGACCGAGTTGCCTAAT	CCGGCTCTTTCAGAATGGGT
Tcf3	ACGAGCTGATCCCCTTCCA	CAGGGACGACTTGACCTCAT
Gata2	CACCCCGCCGTATTGAATG	CCTGCGAGTCGAGATGGTTG
Gata3	CTCGGCCATTCGTACATGGAA	GGATACCTCTGCACCGTAGC

Myst3	AGGAGGAGGAAGAGCCAGAG	ATTGTGGTGTTTCCGCTTGC
Kdm1	ATGATGTCTGGGAAACAGGC	AATGAAAGTGAGCCGGAAGA
Dnmt1	GGTCAACGAGGCAGACATCA	TTCACCACAGCTTCCTCGTC
Dnmt3a	GAGGGAACTGAGACCCCAC	CTGGAAGGTGAGTCTTGGCA
Dnmt3b	CAGACAGGGCAAAAACCAGC	TTGGACACGTCCGTGTAGTG
Ezh2	CGACTGCATTCAGGGTCTTT	ACTTCTGTGAGCTCATTGCG
Bmi1	ACCTGGAGAAGAAATGGCCC	CACTTTCCAGCTCTCCAGCA
Phf20	CTACCAGAAGAAGCCCCTGC	AGCCAGCTCTCCAACACATC
Tet1	GCGTCCTTTCTCTGGTGTCA	TGCTTCCGTTGTGCATGTTG
Tet2	ACAAATGACAGCACAACCGC	CTGAAGGTGCCTCTGGAGTG
Tet3	TCCGGGAACTCATGGAGGAT	TGTGTCTTCGGATCACCCAC
Bmprla	ACAGGAGGAATCGTGGAGGA	GTTCCAGCGGTTAGACACGA
Stat3	GGATCGCTGAGGTACAACCC	GTCAGGGGTCTCGACTGTCT
Ctnbl1	TGAAGAGGCTGAAGGCCAAG	GAAGCACGTCAATGCCATCC
Nes	AGCAGGAGAAGCAGGGTCTA	TTGGGGTCAGGAAAGCCAAG
Wnt1	AACCACAGTCGTCAGAACCG	TATGTTCACGATGCCCCACC
Gsk3b	GCCACAGCAGCCTCAGATAC	GCTGTTCAGGTGGAGTTGGA
Csnk2a1	TTGGGCAGACACTCCCGTAA	TCAGGGCTGACAAGATGCTG
Lif3	ACACTTTCCGGGTTCGTTGT	TGAAGGAGTGGCTTCTGTGG
Hes	GGCCTCTGAGCACAGAAAGT	GGAATGCCGGGAGCTATCTT
Jag1	ATACACGTGGCCATCTCTGC	AGTGAGCTGTTTCCATCCCG
Notch1	AGTGAGCTGTTTCCATCCCG	ATTCTTCAGGGGCTTGAGGC
Fgf4	CAACTATGAGCGCTTCGTGC	AAGTAGCGGCGATAGACAGC
Fgf5	GAAAACTCCATGCAAGTGCCA	CCACTCTCGGCCTGTCTTTT
B-Catenin	CGCCTTTGCGGGAACAGGGT	ATGCGCACGCCCTCCACAAA
Ink4a	CGTACCCCGATTCAGGTGAT	TTGAGCAGAAGAGCTGCTACGT
Arf	GCCGCACCGGAATCCT	TTGAGCAGAAGAGCTGCTACGT
p21	GTGGGTCTGACTCCAGCCC	CCTTCTCGTGAGACGCTTAC
p53	TCCGACTGTGACTCCTCCAT	CTAGCATTCAGGCCCTCATC
Bub1	CAAAAGCATTTGCTTCTTTCC	TGAAGAACTGAGAGCCCAGAA
Cdc20	GGAGACCAGAGGATGGAGC	CAGGAGGAGGAACCAGTGAC
Mad2l1	CGTGGCCGAGTTTTTCTCATT	ATGAGCTCGGGGTCAGTAGT
Ccnf	GATTCGGATCCCTACCGTGG	CTCACAGAGGAAGCTGCACA
Cdh1	CAACGATCCTGACCAGCAGT	TGTATTGCTGCTTGGCCTCA
Cldn3	GAGTGCTTTTCCTGTTGGCG	TCCCTGATGATGGTGTTGGC
Cldn4	CTTCATCGGCAGCAACATCG	ACTGCATCTGACCTGTGCTC
Cldn7	GTACGAGTTTGGACCTGCCA	ACGCAGCTTTGCTTTCACTG
Cldn11	TATTCTGCTGGCTCTCTGCG	CGAGTAGCCAAAGCTCACGA

Epcam	CATTTGCTCCAAACTGGCGT	TTGTTCTGGATCGCCCCTTC
Crb3	TAAACTCATGGCGACCCCAG	AAAGGGTCCGGTGCTGTTAG
Snail	AAGATGCACATCCGAAGCCA	ATGGCTTCTCACCAGTGTGG
Slug	CGAACCCACACATTGCCTTG	AGGCTTTTCCCCAGTGTGAG
Zeb1	AATGACTCTGACTCCACGCC	CGTGAGGCCTCTTACCTGTG
Zeb2	CTGTTTCTTCGCCTCCACCT	GCAGGCTCGATCTGTGAAGT
Ocln	TGGCAAAGTGAATGGCAAGC	GGAATCTCCTGGGCCACTTC
N-Cadherin	CCTCCAACGGGCATCTTCAT	CTCTCAAGTGAAACCGGGCT
Tgfbr1	CTGCTGACCCCCACTGATAC	AGCCCTGTATTCCGTCTCCT
Tgfbr2	CACCAAAGTCCTCAGCCTGT	GATCCTGGGACACACAGCAA
Tgfbr3	TTACTGCTTCCGCAACCTGG	AGGTTCGTGGACCCATTTCC
Tgfb1	GCTGTGAGGCCTTGAGAGTG	TCCCACGGTGTTTCAGTTACA
Tgfb2	ACGTTCCCAAGTCGGATGTG	GCTGGCCATGACATCACTGT
Tgfb3	TTACTGCTTCCGCAACCTGG	AGGTTCGTGGACCCATTTCC
Rbpj	ATGCCCTCCGGTTTTCCTC	GGACAAGCCCTCCGAGTAGT
Usp26	CTCAAGTCCAGATGTGGAGTGC	CTGGTCTTCGCCATAGGTTTG
Trim8	AGGGACACTCGGTGTGTGA	TGTCTGCCGCAAGTCTTCATC
Trim35	TTCCGGGCCAAGTGTAAGAAC	CCAAGTCGTTTGCACCTCA
Trim46	GGTGAGGATATGCAGACCTTCA	TTGTGGGTACAAGGCAGCAC
Cbx4	GATATCCCATCAGCTGCTCC	GGCTGGTCCCCCAAATATAA
Cbx6	CTCGGCTTGACAGAGAAATG	AGGGAACGTGAGCTGTATGG
Cbx7	AGGGTCCAAGATGTGCTCC	CGGAAGGGCAAAGTTGAATA
Ring1a	TGGGACACATGAGCTCAGAA	CTCTATGAGCTGCACCGGAC
Pcgf2	ATTTTAATCCGTGTGGTCCG	CGCCTGATCCTGTTCAAGTT
**ChIP-qPCR (Mouse)**		
Oct4 promoter	AAGCTGAGGCACAGCAAACTGAAG A	GGAGGATGCATGGGAATAGGGC
Nanog promoter	ATCCACCTGCCTCTGCCGCCTAA	GCATTGGTGTTTTGCCTGCATGG
Sox2 promoter	CTTCCAAATGCATTTCCCTGTCGTC	TGGGGAAACTGCTGAGCCTTCAG
Cbx4 promoter	CCCCTTTCTGCTGAGGTTCT	CAGTCTCTGGGCATTTAGGC
Cbx6 promoter	GCCTAAATGCCCAGAGACTG	GGCCTCTGTTTCTGCTTGTG
Cbx7 promoter	TTATTTAGAAGGGGCTTCCTTTG	GGGTGTTTGTAAAGGAGGGATAG
Usp26 promoter	GAATTGGTAGGAAAGAACTC	AAGATTGACAGGGATAATATTTAC
**Cloning primers**		
Human Usp26-C304S	TGTAACACTGCATTCATATAAGCG GTGTTTCCCAAATTGGGGAG	CTCCCCAATTTGGGAAACACCGCTTAT ATGAATGCAGTGTTACA
Human Usp26-Δ297-312	GAGAAAATATGCCACGGCCTACTT TCAATCCCATCG	CGATGGGATTGAAAGTAGGCCGTGGC ATATTTTCTC
**sgRNA target sequences**		

Mouse Usp26	GTAATTCTGGTCTTCGCCAT	
Mouse Cbx4	CCACGCGCAGGATCGTATCG	
Mouse Cbx6	TCCGCGCCTCACGCTGAAGT	
Human Cbx4	TGGCGAGCACGTCTTCGCGG	
Human Cbx6	CATCAAACGGCGGATCCGAA	
Human Ring1a	ACTCCTTGTTCCCGCTCCGT	
**shRNA target sequences**		
Mouse Usp26	GAACTCTGACTACTATCCT	
Human USP26	TATCCCACTTTGTGTAACC	
Mouse Usp1	TACTTTCACTAGGTATGAC	
Mouse Usp2	TCATAGAAGAGCAAATAGG	
Mouse Usp3	TCATGAAACAAGTGTTGCC	
Mouse Usp4	TTCAGCAATTTATTCCAGG	
Mouse Usp5	TAGTGGAGAACTCAGGGTG	
Mouse Usp7	TTTATTATCTTCAGCACTG	
Mouse Usp8	ATCCAACTAGCAGTGACTC	
Mouse Usp9	TCTATTGTCGTACTATCTG	
Mouse Usp10	TAACTCTGCAATCTTTATG	
Mouse Usp11	TGTCTGGTGCAACACTTGT	
Mouse Usp12	AACTATTGCAATATAATGG	
Mouse Usp13	ATAGCTACGATTTCCTCAG	
Mouse Usp14	ATCATCATCAAATTTGATC	
Mouse Usp15	TTATAGCGGCAAGGACCTG	
Mouse Usp16	AACATAATCAACCACTTGG	
Mouse Usp18	TATTATTCTCGTTGAAATG	
Mouse Usp19	TTACTTTCCAGGTTTGCTC	
Mouse Usp20	TTCTTGAGCACATACGACG	
Mouse Usp21	TTCTTGAGCCTTTCATCTG	
Mouse Usp22	AAATACCTCCGTACATCAG	
Mouse Usp24	TTTAGCATCCAAGGAAACC	
Mouse Usp25	ATCTTCAAGATCATGTGAC	
Mouse Usp28	TAGCTAAGGTTTCTATCCG	
Mouse Usp29	AAATCCAGATGATACAGTC	
Mouse Usp30	ACAGTTGTCACACACAACG	
Mouse Usp31	TATTCTGAAGTTTCATACG	
Mouse Usp32	ATTGAAATGCAGCCCTTTG	
Mouse Usp33	TATAGACTGCAGGAAATTC	
Mouse Usp34	TAGGTCAGCCATATTACGA	

Mouse Usp35	TTCTCTTCTTCATGAAGCC	
Mouse Usp36	TGTTCCCTGAGAATCACAG	
Mouse Usp37	TATCATCTTCTGCAAAGCC	
Mouse Usp38	ATAGTAAACTTCTGAACAG	
Mouse Usp39	TTACCATCTTCAGCCACAG	
Mouse Usp40	ATCCTCTTCAAATAGGTTC	
Mouse Usp42	TAATCTCCAACGTTATGTC	
Mouse Usp43	TCTTTCGACCTAAGGAAAG	
Mouse Usp44	TTCCAATGATTCAGAGATC	
Mouse Usp45	TTATTTGGGACATTTAGTG	
Mouse Usp46	TATATTAGAGACCAGTAGC	
Mouse Usp47	AAGAATATCACTGTCTACC	
Mouse Usp48	TCTCTCAGGGAATAAAGAC	
Mouse Usp49	ACATGTTTGCATCTATCCA	
Mouse Usp50	TGAACAGAAGTATCTGGGA	
Mouse Usp51	ATGCAACACATCCAATATG	
Mouse Usp52	ACAGTTGTCACACACAACG	
Mouse Usp53	TTCAACTTCATTGCACAGG	
Mouse Usp54	ATAGTTCAGACTGCTTGGC	
Mouse Ring1a	ATTTGTTGTTGGGAGGAGG	
Mouse Bmi1	TCATTCACCTCTTCCTTAG	
Mouse Cbx8	TTTCACGAGATATTCCATG	
Mouse Cbx2	TTCTTTCTAATGGGATCCT	
Mouse PCGF2	AGGTGTAGTATTCCTTCAG	
Mouse Cbx4	TTATATTCCCAAAGAAGGG	
Mouse Cbx6	TCTGTGACAACTACATTGG	
Mouse Cbx7	TCCGGATGCTCTCCACCGC	
Mouse Ring1b	AAGACATTAAGTCTGACTG	
Mouse PCGF1	AGTCGGTGACACAGAACCC	
